# Integrated 16S rRNA Gene Sequencing and Metabolomics Analysis to Investigate the Important Role of Osthole on Gut Microbiota and Serum Metabolites in Neuropathic Pain Mice

**DOI:** 10.3389/fphys.2022.813626

**Published:** 2022-02-07

**Authors:** Ruili Li, Fan Wang, Shajie Dang, Minna Yao, Wei Zhang, Jingwen Wang

**Affiliations:** ^1^Department of Pharmacy, Xijing Hospital, Fourth Military Medical University, Xi’an, China; ^2^Department of Anesthesiology, Shaanxi Provincial Cancer Hospital, Xi’an, China

**Keywords:** osthole, gut microbiota, serum metabolomes, neuropathic pain, 16S rRNA gene sequencing

## Abstract

Accumulating evidence suggests that neuropathic pain (NP) is closely connected to the metabolic disorder of gut microbiota, and natural products could relieve NP by regulating gut microbiota. The purpose of this study is to investigate the important regulatory effects of osthole on gut microbiota and serum metabolites in mice with chronic constriction injury (CCI). Mice’s intestinal contents and serum metabolites were collected from the sham group, CCI group, and osthole treatment CCI group. The 16S rRNA gene sequencing was analyzed, based on Illumina NovaSeq platform, and ANOVA analysis were used to analyze the composition variety and screen differential expression of intestinal bacteria in the three groups. Ultra-high-performance liquid chromatography-quadrupole time of flight-tandem mass spectrometry (UHPLC-Q-TOF-MS) was used for analyzing the data obtained from serum specimens, and KEGG enrichment analysis was used to identify pathways of differential metabolites in the treatment of neuralgia mice. Furthermore, the Pearson method and Cytoscape soft were used to analyze the correlation network of differential metabolites, gut microbiota, and disease genes. The analysis results of 16S rRNA gene sequencing displayed that *Bacteroidetes*, *Firmicutes*, and *Verrucomicrobia* were highly correlated with NP after osthole treatment at the phylum level. *Akkermansia*, *Lachnospiraceae_unclassified*, *Lachnospiraceae_NK4A136_group*, *Bacteroides*, *Lactobacillus*, and *Clostridiales_unclassified* exhibited higher relative abundance and were considered important microbial members at genus level in neuralgia mice. Serum metabolomics results showed that 131 metabolites were considered to be significantly different in the CCI group compared to the sham group, and 44 metabolites were significantly expressed between the osthole treatment group and the CCI group. At the same time, we found that 29 differential metabolites in the two comparison groups were overlapping. Integrated analysis results showed that many intestinal microorganisms and metabolites have a strong positive correlation. The correlation network diagram displays that 10 genes were involved in the process of osthole alleviating NP through a metabolic pathway and gut microbiota, including IGF2, GDAP1, MYLK, IL18, CD55, MIR331, FHIT, F3, ERBB4, and ITGB3. Our findings have preliminarily confirmed that NP is closely related to metabolism and intestinal microbial imbalance, and osthole can improve the metabolic disorder of NP by acting on gut microbiota.

## Introduction

Neuropathic pain (NP) is caused by primary nervous system injury and dysfunction, which seriously affects the quality of patients’ life (Barragan-Iglesias et al., 2014; Alonso-Castro et al., 2018). At present, treatment options for NP include nerve block with anesthetics, opioid receptor antagonism, nerve damage, etc. (Garnier et al., 2003; Mendlik and Uritsky, 2015). Although these treatments have achieved sound analgesic effects, they also have various insurmountable side effects, such as pain sensitivity, addiction, tolerance, nausea, and constipation (Skolnick, 2017). Improving the clinical therapeutic effect and reducing the toxic and side effects of drugs have become the goal of the joint efforts of medical and health workers, and primarily through the regulation of diseases by human endogenous substances will achieve two times the result with half the effort. Recent studies have shown a link between intestinal microorganisms and NP and gut microbiota changes in patients with NP (Guo et al., 2019; Toh et al., 2020). The two-way signal transmission between intestinal microorganisms and the brain through neural, incretion, immune, and metabolic pathways are called the gut-brain axis, which involves many human organs such as the brain, gland, intestine, immune cells, and intestinal microorganisms (Cryan and Dinan, 2012; Montiel-Castro et al., 2013). Therefore, regulating the gut microbiota may be a practical and feasible strategy for treating NP.

How does intestinal flora affect the occurrence and progression of neuralgia? It is known to all that metabolites are an essential link between intestinal flora and NP. On the one hand, intestinal microorganisms activate stress response through the hypothalamic pituitary adrenal axis and they affect intestinal permeability, motility, and mucus production (Yan and Kentner, 2017; Afifa et al., 2018). Metabolites (e.g., LPS, SCFA, peptidoglycans, trimethylamine, and secondary bile acids) in plasma deliver proinflammatory cytokines (e.g., TNF-α, IL-1β, and IL-6), chemokines (e.g., CCL2 and CXCL1), anti-inflammatory cytokines (e.g., IL-4) by activating non-neuronal cells (such as immune cells, astrocytes, or microglia), or neuropeptides (e.g., opioids), and these inflammatory factors directly cross the blood–brain barrier (BBB) and cause pain sensitivity (Varatharaj and Galea, 2017; Zhu et al., 2018). When intestinal permeability increases, the level of proinflammatory factors in the plasma increases, and the severity of pain increases (Piche et al., 2009; Ernberg et al., 2018). On the other hand, intestinal microorganisms change the behavioral and cognitive functions by regulating neurotransmitters release, including norepinephrine, γ-aminobutyric acid, brain-derived neurotrophic factor, and dopamine. Metabolites directly activate pain-related receptors or ion channels (such as TLRs, TRP channels, GABA receptors, and acid-sensing ion channels) in primary neurons to regulate neuronal excitability (Ji et al., 2014, 2016; Chow and Gulbransen, 2017; Chiu, 2018). Therefore, it may alleviate the occurrence of NP by regulating intestinal microorganisms and affecting metabolites’ balance. However, the endogenous mechanism of metabolite changes includes how metabolites are produced, their upstream pathways, and proteins are, and which protein plays a role is unknown until now The relationship between intestinal microorganisms and their metabolites in neuralgia deserves further study.

More and more researchers have paid attention to environmental factors (such as nutrition and natural products) to treat nervous system diseases through the intestinal brain axis (Viaud et al., 2013; Mukaida, 2014; Vazquez-Baeza et al., 2018). Natural products or medicines can affect intestinal flora and change its activity, resulting in different bioactive metabolites. Osthole, a coumarin compound, was extracted from the natural product *Angelica biserrata Yuan et*. Some reports have found that osthole possess a variety of pharmacological activities, including anticancer (Dai et al., 2018; Park et al., 2019), antioxidant (Shokoohinia et al., 2014), and anti-inflammatory (Xu et al., 2018). Research revealed that osthole alleviated nociceptive responses by suppressing ASIC3 in rat dorsal root ganglion (He et al., 2012). Our previous study showed that osthole alleviated mechanical and thermal pain sensitivity in mice with chronic constriction injury (CCI) and played a role in the treatment of NP through the P2Y1-receptor-dependent JNK signaling pathway (Li et al., 2020). It is required to carry out a comprehensive research about relieving neuralgia of osthole, especially the intestinal microbial metabolic pathway. In the present study, we employed integrated 16S rRNA gene sequencing and metabolomics to analyze the crucial targets and mechanisms of osthole in treating NP because of gut microbiota and metabolomics for the first time. Furthermore, the relationship between gut microbiota, metabolites, and NP disease targets was analyzed to obtain the molecular targets of NP that may participate in the intestinal brain axis. This study will help us understand the process of osthole relieving NP from a new perspective, find the biomarkers of osthole in the treatment of NP, and provide a sufficient theoretical basis for the clinical practice of osthole.

## Materials and Methods

### Drug and Reagents

Osthole (>98%, CAS: 484-12-8) was obtained from Yuanye Biotechnology Co., Ltd. (Shanghai, China). Phusion Hot start flex 2× Master Mix was supplied by Shanghai Yitao biological Instrument Co., Ltd. (Shanghai, China). DL2000 DNA Maker was obtained from Takara Bio INC. (Kyoto, Japan). Gene color was purchased from Beijing Jinboyi Biotechnology Co., Ltd. (Beijing, China). Qubit dsDNA HS ASSAY Kit was obtained from Invitrogen and Life technologies company (CA, United States). The 50× TAE buffer was obtained from Shanghai Shenggong Bioengineering Co., Ltd. (Shanghai, China). Biowest Agarose G-10 and AMPure XT beads were purchased from BIOWEST (Loire Valley, France) and Beckman Coulter (CA, United States) company; Water DNA Kit (200), Soil DNA Kit (200), and Stool DNA Kit (200) were all purchased from OMEGA bio-tek, Inc. (Norcross, GA, United States). Methanol, acetonitrile, and formic acid are chromatographic reagents and are available in the market.

### Animal and Experimental Design

**FIGURE 1:**
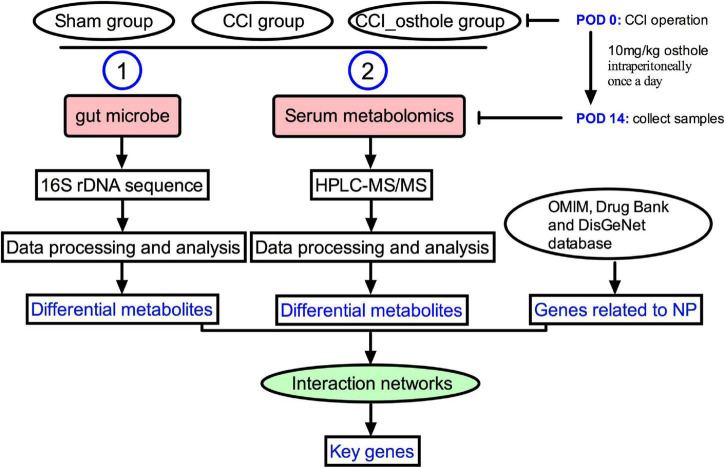
The experimental flow chart. The mechanism of osthole against chronic constriction injury (CCI) neuralgia mice was analyzed by 16S rRNA and metabolomics.

Healthy C57BL/6 mice (6–8 weeks) were obtained from the animal experiment center of the Fourth Military Medical University (approval number: XJYYLL-2015612). This experiment was carried out with consent from the Chinese Food and Drug Administration (cFDA). The best effort was contributed to minimizing the number of animals used and their suffering. Mice were placed in a cage at 22 ± 2°C, 45–75% humidity, and 12/12 h of alternating light. They were fed and drank freely for 1 week. A total of 18 mice were randomly divided into 3 groups (*n* = 6): Sham group, CCI group, and treatment of CCI model with osthole (10 mg/kg). The sham group and CCI group received an equal volume of vehicles. Osthole was administered intraperitoneally after surgery from post-operative day (POD) 0 to POD 14. On POD 14, the intestinal contents of the mice were collected and blood was harvested from the orbital sinus. The experimental flow chart is shown in Figure 1.

### Establishment of Chronic Constriction Injury Mice Model

The L5–L6 CCI model of neuropathy was established as previously reported (Park et al., 2013). Briefly, the skin was cut about 1 cm below the femur parallel to the femur on the mice’s left hind limb. The muscle was bluntly separated through the biceps femoris space and the sciatic nerve was exposed. Then, the 7 mm nerve at the main part of the sciatic nerve was dissociated before it was divided into three branches. At 2 mm above the beginning of the nerve (the bifurcation of the three branches), three sciatic nerves were ligated with 4.0 chromium containing catgut, with an interval of about 1 mm, so that the length of the ligated nerve is about 4–5 mm. Attention was paid to the tightness of ligation, subject to slight muscle twitch during knotting. The muscle fascia, subcutaneous tissue, and skin were intermittently rinsed with local normal saline and sutured. After the operation, the mice were put into the cage and fed freely in a warm and clean environment. The sham operation group was the same as the model group except that the sciatic nerve was not ligated. The operation was performed by the same person.

### 16S rRNA Amplicon Sequencing of Intestinal Contents Samples

The mouse intestinal contents samples were collected on POD 14 in a super clean platform. Following the instructions of E.Z.N.A.^®^Stool DNA Kit, the V3–V4 regions of bacterial 16S rRNA gene was PCR-amplified using barcoded conventional primers (341F 5′-CCTACGGGNGGCWGCAG-3′ and 805R 5′GACTACHVGGGTATCTAATCC-3′) under conditions as follows: initial denaturation at 98°C for 30 s, 32 cycles of denaturation at 98°C for 10 s, annealing at 54°C for 30 s, extension at 72°C for 45 s. The polymerase chain reaction (PCR) products were examined by gel electrophoresis and purified by Ampre XT beads. The amplified products were analyzed by Agilent 2100 Bioanalyzer (Agilent, United States) and Illumina kit, and the size and quantity of the amplified library were determined. The NovaSeq PE250 tool was used for sequencing (Cheng et al., 2017; Zhang et al., 2018).

### Illumina Sequencing Data Analysis

The samples were sequenced on the Illumina NovaSeq platform. According to the unique barcode of the samples, the paired-ends reads were assigned, and the barcode and primer sequences introduced during database construction were dislodged. Paired-end reads were merged using FLASH. According to fqtrim (v0.94), raw reads data were filtered under specific filtering conditions. Data dereplication was carried out by DADA2, which is equivalent to clustering with 100% similarity, to get the feature table and feature sequence. The relative abundance of each sample was used to normalize the feature abundance. Alpha diversity and beta diversity were used to analyze species diversity within and between the samples, respectively. Alpha diversity was measured through indexes of Chao1, observed species, goods coverage, and Shannon. QIIME2 software was used to analyze all indices. The feature sequences were treated based on SILVA DB (Brandon-Mong et al., 2020).

### Extraction and Detection of Plasma Metabolites in Mice by High Performance Liquid Chromatography-Tandem Mass Spectrometry Analysis

A total of 120 μl of 50% methanol buffer was added in 20 μl serum obtained by centrifugation (3,000 rpm, 15 min) to extract metabolites. The metabolite mixture was mixed sufficiently and allowed to stand at 23°C for 10 min. After storing at −20°C overnight, proteins in the samples were subsided and centrifuged at 4,000 × *g* for 20 min and then analyzed by LC-MS. Besides, the quality control (QC) sample was got based on the 10 μl extraction mixture. The sample was analyzed by the UPLC system (SCIEX, United Kingdom). During the experiment, an ACQUITY UPLC T3 column was chosen to separate the samples. The mobile phase is composed of solvent A and B under a flow rate of 0.4 ml/min; 100% solvent B for 7–8 min; and 5% solvent B for 10 min. The metabolites detection was conducted by The TripleTOF 5600 Plus system. Each sample collected signals of two kinds of ion mode. The shielding pressure of the ion source is 30 psi (pounds per square inch), the ion source gas 1 (auxiliary gas) and gas 2 (sheath gas) pressure was set to 60 psi. The interface temperature was 650°C. The ion spray floating voltage was 5 kV in positive-ion mode. In the process of analyzing the obtained data, the MS data were acquired in the IDA mode. In an acquisition cycle, the TOF mass range is 60–1,200 Da. Then, the first 12 signal ions with a positive charge (with a negative charge in negative ion mode) and a signal accumulation intensity of more than 100 per second are selected from the primary map for secondary fragmentation scanning. The whole acquisition cycle takes 0.56 s. The detector of the mass spectrometer has four channels, and the frequency of pulsed RF is 11 kHz, whereas the frequency is 40 GHz. The particle signals information was recorded in four channels a total of four times, and then combined and converted into data. The dynamic exclusion time of the scan is set to 4 s. The instrument accuracy shall be corrected every 20 samples. At the same time, scan QC products every 10 samples. The quality difference between QC is used to correct the systematic error of the whole batch of experiments (Chen et al., 2019; Feng et al., 2019).

### Data Processing of Non-targeted Metabolomics

The acquired LC-MS data pre-treatment was performed using XCMS software. In the research process, in order to facilitate processing, the original data is transformed into mzXML format, and then the statistical analysis is carried out by using the tools related to XCMS and metaX in the software, and the graphical results are given. The retention time and *m/z* parameters were used to identify the parent ions. The intensity of each peak was recorded and determined and then processed to generate a matrix including peak index, observed value, and ionic strength. Then, the obtained information was matched with the database to provide support for subsequent analysis. The KEGG and HMDB databases were used for labeling, and the molecular weight data and related threshold data were matched. On this basis, the metabolites were annotated. The peak intensity data was pre-processed by the following methods: by removing the corresponding characteristics of <50% QC samples and then carrying out appropriate extrapolation through the k-nearest neighbor algorithm. On this basis, the corresponding missing peak values were determined to meet the relevant requirements of analysis quality. PCA was performed on the processed data set to determine the abnormal data. Based on QC, certain correction processing was carried out, and on the basis of matching, the drift of belief signal strength is suppressed as much as possible. The deviation of metabolic characteristics in all QC samples was determined and then the samples with obvious deviation were removed. Before the analysis of sample data, it was standardized and applied to the probability quotient method in the normalization process. Then corresponding batch correction through QC samples to provide support for subsequent sample analysis was carried out. The Student’s *t*-test was conducted for the difference of results within the group, and the appropriate metabolites were determined based on the appropriate adjustment of multiple tests by FDR. In the research process, the variables were supervised by metaX, and the differences between the variable groups were analyzed by discriminant analysis. VIP cutoff was set to 1.0 (Yang et al., 2019).

### Statistical Analysis

The gut microbiota and metabolites (*n* = 6) of adult C57BL/6 mice (weight 18–22 g) were randomly obtained. *N* represents the number of animals in each group. Changes in values were determined by ANOVA. The differences between groups were analyzed by one-way analysis of variance, and then Bonferroni post-test. The data were analyzed by SPSS (IBM SPSS statistics v19.0), and all statistics were created using GraphPad prism 6.0.

## Results

### Effect of Osthole Intervention on Gut Microbiota Diversity in Chronic Constriction Injury Mice

**FIGURE 2:**
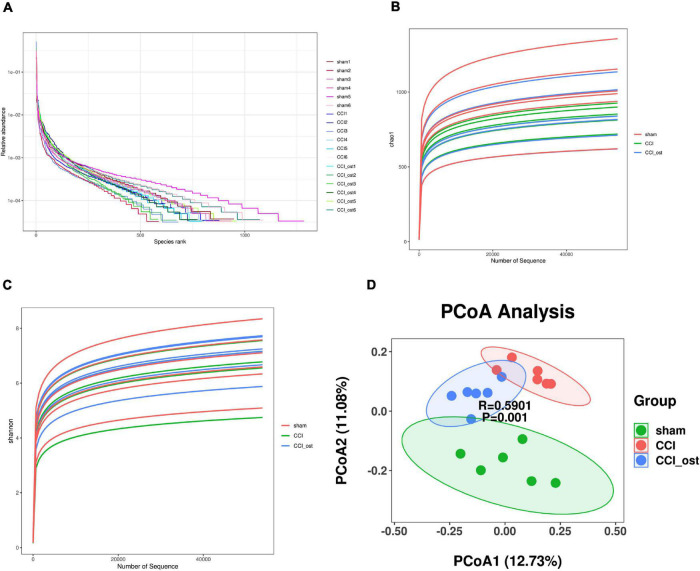
Effects of osthole on the diversity of gut microbiota. **(A)** The rank abundance curves. **(B)** The Chao1 indices were used to estimate the diversity of the gut microbiota. **(C)** The Shannon indices were used to estimate the diversity of the gut microbiota. **(D)** Principal coordinates analysis (PCoA) diagram illustrating the difference in microbial composition among the three groups.

The effect of osthole on gut microbiota composition in CCI mice was conducted with 16S rRNA sequencing using Illumina NovaSeq as a sequencing platform. For improving the reliability of the data, the species resolution, and the reliability of the results, we obtained the representative sequence using QIIME 2 software. The rank abundance curves (Figure 2A) reflect the fact that all microbial communities are also very similar in structure, showing a comparable trend in both grade frequency and abundance in the same group of samples. The content of most of the microorganisms in the intestinal contents of mice was less. According to the alpha diversity analyses, the Chao1 and Shannon diversity indices curves (Figures 2B,C) of the sequencing number of all samples reached the platform stage, indicating that most diversity has been captured. We have got a total of 9,512 representative feature sequences. A sample in the sham group contained the most feature sequences, 1,283, and a sample in osthole treatment CCI group contained the least feature sequences, 585 (Supplementary File 1). To evaluate the difference in gut microbial composition among the three groups, we performed the beta diversity and principal coordinates analysis (PCoA). The PCoA plot based on unweighted unifrac distances results showed that the CCI group exhibited significant separation from the other two groups markedly (Figure 2D), indicating a significant difference of gut microbiota composition in the CCI group compared to the sham group, whereas osthole treatment reduced the difference. The value on the axis records the percentage of results interpreted by each dimension. It can be seen from the figure that PCoA1 and PCoA2 accounted for 12.73 and 11.08% of the total analysis results, respectively.

### Regulation of Osthole on the Structure Constitutions of Gut Microbiota in Chronic Constriction Injury Mice

**FIGURE 3:**
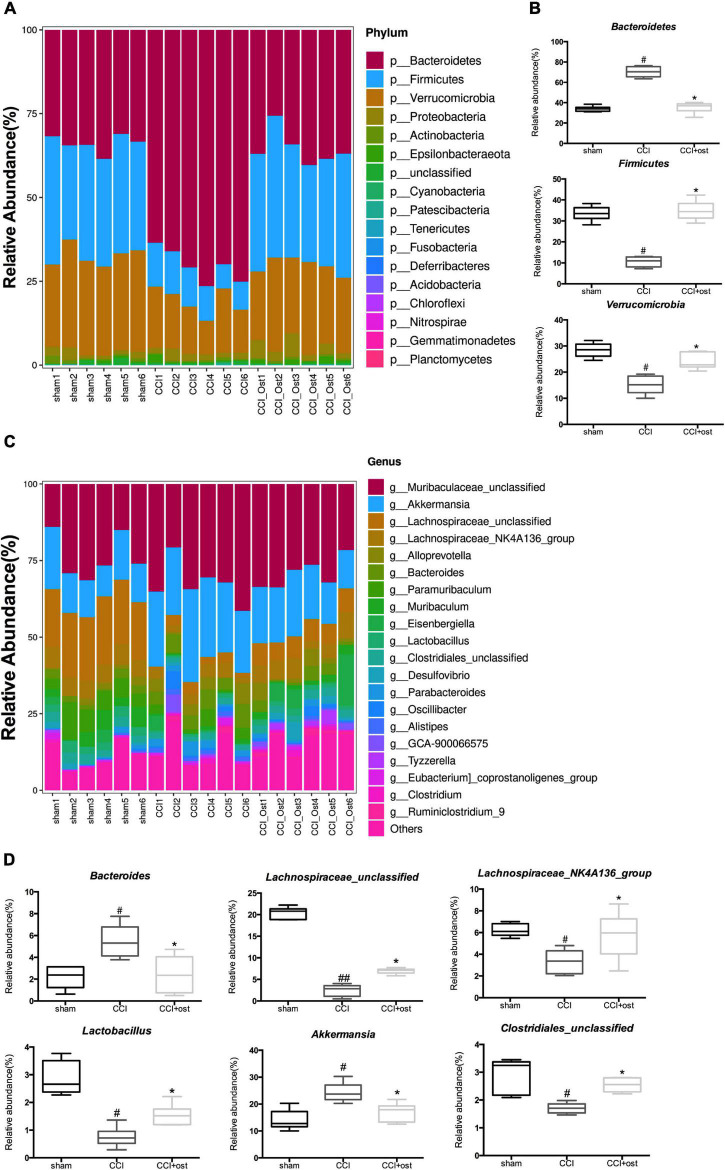
Effects of osthole on the structure of gut microbiota. Based on the quantitative data, the relative abundance of gut microbiota was displayed in a stacked bar plot, including the dissimilarity of gut microbiota in phyla level **(A)** and in phyla level **(C)**. **(B)** The gut microbiota differed significantly among the groups in phyla level **(B)** and in phyla level **(D)**. ^##^*p* < 0.01, ^#^*p* < 0.05 compared with sham group; *p < 0.05, compared with chronic constriction injury (CCI) group, analysis of variance (ANOVA) followed by Bonferroni *post-hoc* test, *n* = 6 mice/group.

To discuss the abundance variation of some specific intestinal bacteria among the three different groups, we selected the top 20 species with the highest abundance to calculate the relative abundance so as to draw the sample relative abundance stacked histogram. At the phylum level, the expression quantity of *Bacteroidetes, Firmicutes, and Verrucomicrobia* was highly correlated with NP (Figure 3A and Supplementary File 2). The expression quantity of *Bacteroidetes* and *Verrucomicrobia* was markedly increased, whereas *Firmicutes* decreased in CCI mice compared to the sham group. Osthole treatment remarkably reduced the quantity of *Bacteroidetes* and *Verrucomicrobia* but improved the quantity of *Firmicutes* in CCI mice (Figure 3B). To classify the microbial members and track their quantitative changes at the genus level. *Akkermansia*, *Lachnospiraceae_unclassified*, *Lachnospiraceae_NK4A136_group*, *Bacteroides*, *Lactobacillus*, and *Clostridiales_unclassified* exhibited higher relative abundance and were considered important microbial members at genus level in neuralgia mice (Figure 3C and Supplementary File 3). The relative abundance of *Akkermansia* and *Bacteroides* were increased in CCI mice compared to the sham group, and osthole treatment altered the distribution of these bacteria in CCI mice. Interestingly, the relative abundance of *Lachnospiraceae_unclassified* and *Lachnospiraceae_NK4A136_group* genera decreased in CCI mice, while they increased after osthole treatment. Simultaneously, we counted the relative abundance of *Lactobacillus* and *Clostridiales_unclassified* that have been reported associated with NP. The results revealed that the expression quantity of *Lactobacillus* and *Clostridiales_unclassified* were all reduced in CCI mice compared to the sham group (Figure 3D). The above results show that osthole regulates the structure of gut microbiota in CCI mice.

### Osthole Treatment Alters Specific Bacterial Taxa in Experimental Groups

**FIGURE 4:**
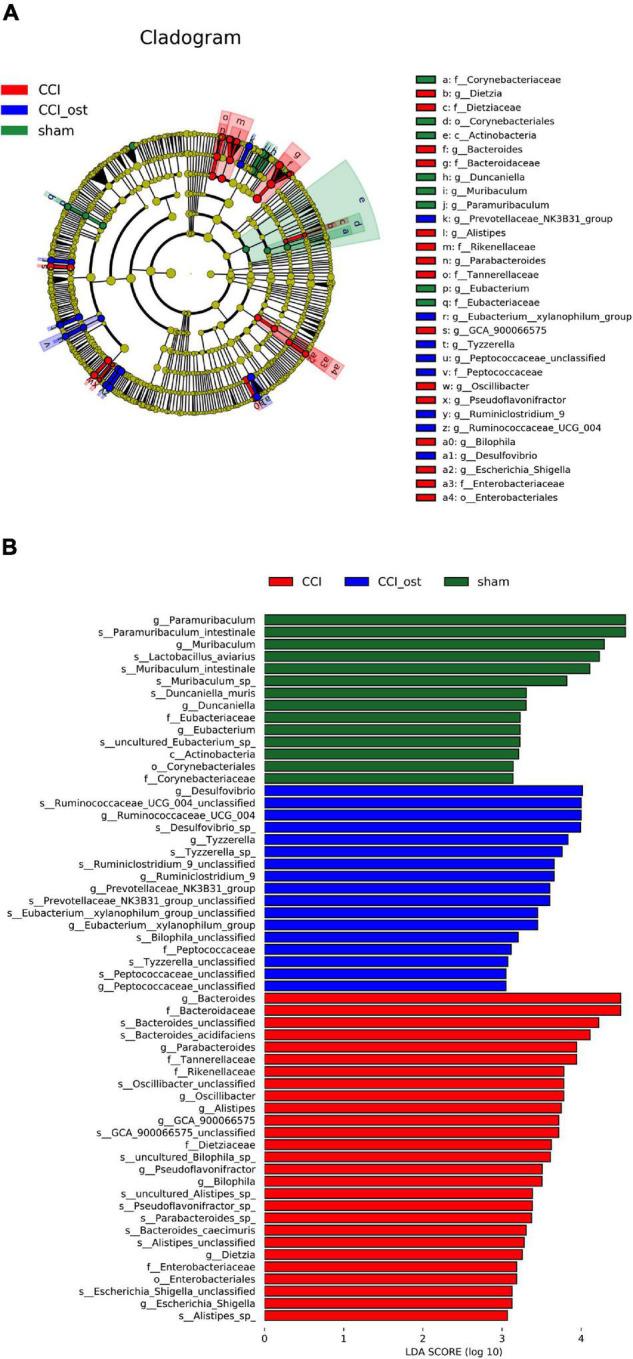
Identification of most specific bacterial taxa by LEfSe analysis. Comparison of gut microbiota composition among experimental groups based on linear discriminant analysis (LDA) effect size (LEfSe) **(A)** and LDA **(B)**. Dot sizes are proportional to the abundance of certain taxa in the taxonomic cladogram, and the greatest differences among groups after LDA using a threshold score of >4.0.

The linear discriminant analysis (LDA) effect size method was also used for contrasting microbiota compositions in the three experimental groups to identify the specific bacterial taxa. The structure composition of gut microbiota emerged with enormous diversity between the groups (Figure 4A). The radiation of different circle layers from inside to outside represents seven classification levels of genera and species of compendium, and each node represents a species classification under this level. The higher the species abundance is, the larger the node show. Four principal genera were found in the sham group, and most of these genera belong to *paramuribaculum*. Concurrently, nine domain genera, among which *Bacteroides* of *Bacteroidaceae* was the most, were identified in the CCI group. Furthermore, seven major genera were detected in the CCI group treated with osthole and most of these genera originated from *Desulfovibrio* of *Desulfovibrionaceae*. The LDA scores also showed that high abundance *paramuribaculum*, *Bacteroides*, and *Desulfovibrio* was a microbiological marker of different treatment groups, respectively (Figure 4B).

### Osthole Treatment Changed Serum Metabolites in Chronic Constriction Injury Mice

**FIGURE 5:**
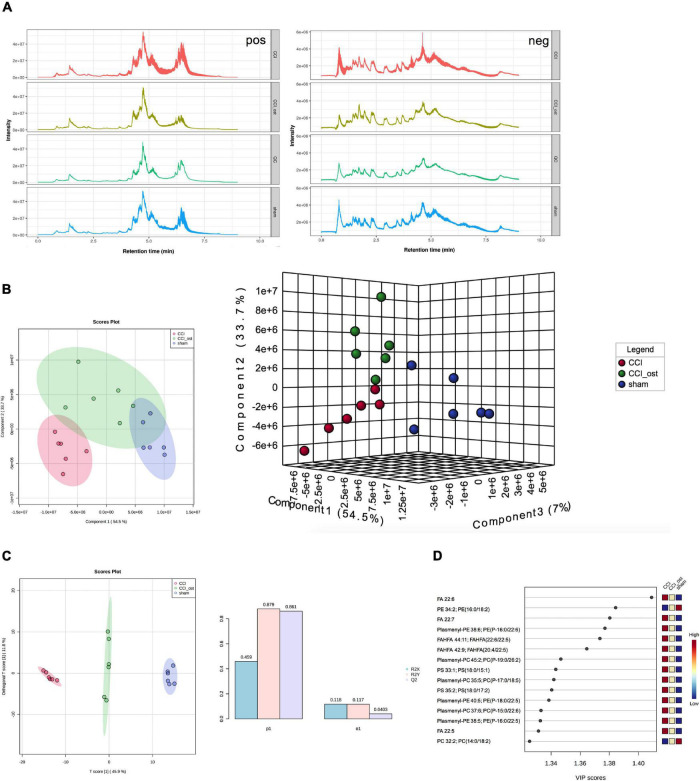
Osthole treatment changed serum metabolites in chronic constriction injury (CCI) mice. **(A)** Total ion flow of mice in each group under positive and negative ion mode. **(B)** PLS-DA 2D and 3D diagram. **(C)** OPLS-DA analysis. **(D)** The variable important for the project (VIP) value of differentially expressed MS2 metabolites.

Ultra-high-performance liquid chromatography-quadrupole time of flight-tandem mass spectrometry (UHPLC-Q-TOF-MS) was used to collect the data of serum samples. The chromatograms of the total ion flow of mice in each group under positive and negative ion mode are shown in Figure 5A. After screening, we obtained a total of 297 significant MS2 metabolites (Supplementary File 4). To investigate the separation among the three different groups, Partial least squares Discriminant Analysis (PLS-DA) and Orthogonal partial least squares discriminant analysis (OPLS-DA) were performed to analyze the ionic strength of the MS2 metabolite we obtained. PLS-DA 2D and 3D diagrams all showed that the CCI group was obviously separated from the sham group, and the samples in each group gathered well. The results showed that the small molecule metabolites in CCI mice changed abnormally, resulting in the sample distribution area of the CCI group being far away from the sham group, and the model replication was successful (Figure 5B). OPLS-DA analysis was consistent with PLS-DA analysis (Figure 5C). The parameters in OPLS-DA were as follows: *R*^2^*X* = 0.459, *R*^2^*Y* = 0.879, *Q*^2^ = 0.861; it follows that the prediction capability of the model established in this study is good. Simultaneously, the sample distribution area showed a callback trend to the sham group after osthole intervention in CCI mice. The variable important for the project (VIP) value obtained by PLS-DA combined with multivariate statistical analysis to screen the differentially expressed MS2 metabolites (Figure 5D). MS2 metabolites with VIP value ≥ 1 included FA 22:6, PE 34:2; PE (16:0, 18:2), FA 22:7, Plasmenyl-PE 38:6; PE (P-16:0/22:6); FAHFA 44:1; FAHFA (22:6/22:5), etc. The above results showed that the CCI model and osthole treatment induced significant metabolic variations.

### Differential Metabolite Identification and Pathway Analysis

**FIGURE 6:**
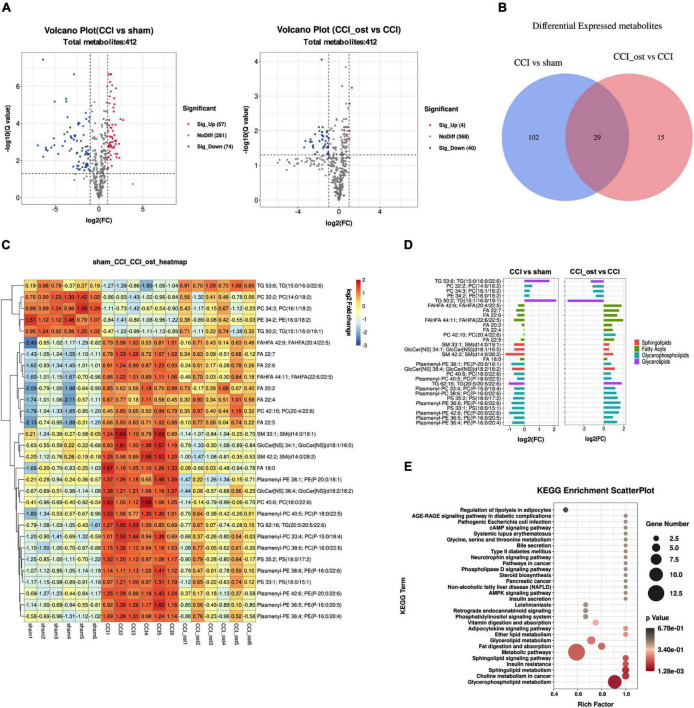
Differential metabolite identification and pathway analysis. Expression of differential metabolites in each comparison group was represented by Volcano plot **(A)**, Venn diagram **(B)**, heat maps **(C)**, and FC histogram **(D)**. **(E)** Kyoto encyclopedia of genes and genomes (KEGG) analysis was used to enrich the pathway of differential metabolites. Data were calculated by the Pearson correlation method after mean centering and unit variance scaling.

Using a lipomic approach, we found potential metabolites that contribute to neuralgia. After sorting and screening, we obtained a total of 412 MS2 metabolites (Supplementary File 5). Based on fold-change (FC) ≥ 2 or ≤ 0.5, *Q* value ≤ 0.05, and VIP ≥ 1, 131 metabolites were significantly expressed between the sham group and CCI group, and 44 metabolites were significantly expressed between the osthole treatment group and the CCI group (Supplementary File 6). Volcano plots reveal that 57 metabolites were raised and 74 metabolites were transferred to a lower unit in CCI mice compared with the sham group. However, 4 metabolites were raised and 40 metabolites were transferred to a lower unit osthole treatment (Figure 6A). The results of the Venn map were basically consistent with those of the volcano map (Figure 6B). At the same time, we found that 29 differential metabolites in the two comparison groups were overlapping. Most of these metabolites belong to glycerolipids, sphingolipids, glycerophospholipids, and fatty acyls. To visualize the changes of metabolites among the three groups, we plotted heat maps and FC histograms (Figures 6C,D). The results showed that the variety of the majority of the metabolites in the CCI group was reversed in the osthole treatment group, demonstrating that osthole treatment could regulate metabolic disorders. KEGG enrichment analysis of differential metabolites showed that multiple pathways are involved in the process of osthole in the treatment of neuralgia, including regulation of lipolysis in adipocytes, AGE-RAGE signaling pathway in diabetic complications, pathogenic *Escherichia coli* infection, etc. (Figure 6E). The metabolites related to these pathways are listed in Supplementary File 7.

### Integrated Analysis of Metabolomics, Gut Microbiota, and Disease Genes

**FIGURE 7:**
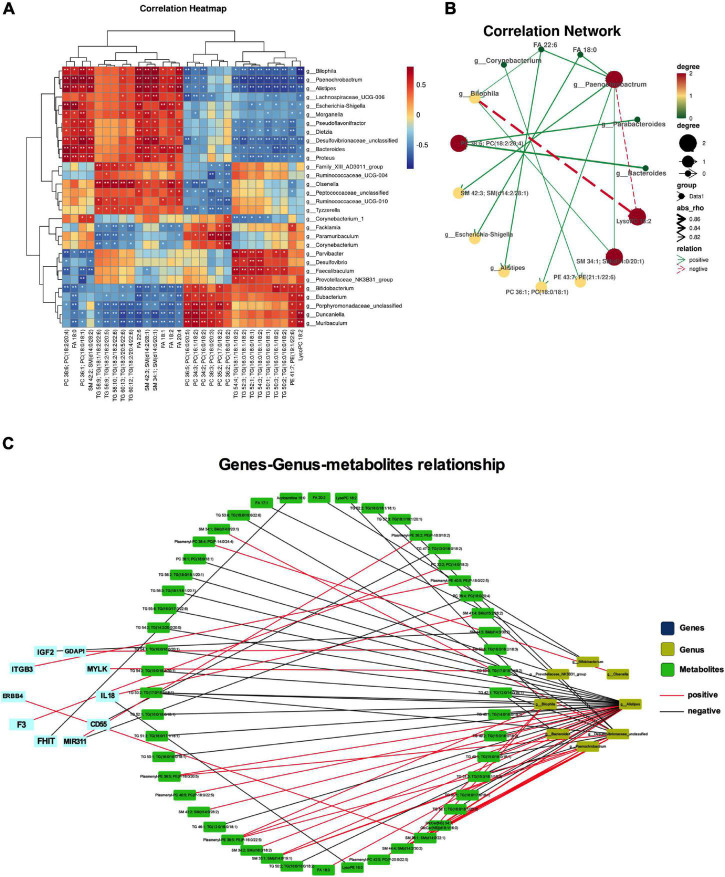
Integrated analyses of metabolites, gut microbiota, and disease genes. Correlation heatmap (*p < 0.05, **p < 0.01) **(A)** and correlation network **(B)** of differential microbiota and metabolites. **(C)** Correlation network diagram of microbiota, metabolites, and disease gene. Data were calculated by the Pearson correlation method after mean centering and unit variance scaling.

Integrated analysis was used to reveal the regulatory relationship between variable intestinal microorganisms and metabolites. Pearson method was used to analyze the relationship between intestinal differential flora and serum differential metabolites in the treatment of NP by osthole. | R| > 0.8 indicates that there is a strong correction. Correlation heatmap (Figure 7A) showed that many intestinal microorganisms and metabolites have a strong positive correlation, such as *Bilophila* and SM 34:1; SM (d14:0/20:1), *Paenochrobactrum* and FA 18:0, PC 36:1; PC (18:0/18:1), FA 22:6, and SM 42:3; SM (d14:2/28:1), *Alistipes* and FA 22:6, *Escherichia-Shigella* and FA 18:0, etc. However, *Paenochrobactrum* and LysoPC 18:2 have a strong negative correlation. The correlation network between intestinal bacteria and metabolites is shown in Figure 7B. To explore how serum metabolites were regulated by upstream genes, the relationship among metabolomics, gut microbiota, and disease genes were analyzed by Cytoscape software. NP genes were obtained from OMIM,^1^ Drug Bank database,^2^ and DisGeNet database.^3^ We inputted “neuropathic pain” as the keyword to get related genes. After sorting out and removing duplicate genes, we obtained a total of 1,012 genes. The gene symbols were unified according to UniProt. Further analysis was conducted in Cytoscape software. The corAndPvalue function of WGCNA in the R package was used to calculate the correlation of genes, metabolites, and microbial genera. The correlation network diagram (Figure 7C) displays that 10 genes were involved in the process of osthole alleviating NP through the metabolic pathway and gut microbiota, including IGF2, GDAP1, MYLK, IL18, CD55, MIR331, FHIT, F3, ERBB4, and ITGB3.

## Discussion

At present, the relationship between intestinal flora and pain regulation has attracted more and more attention from clinicians with the progress of medical science and technology. A large number of experimental data showed that intestinal microorganisms and their metabolites are inextricably linked with the occurrence and development of NP (Shen et al., 2017; Yang and Chiu, 2017; Chiu, 2018; Defaye et al., 2020). The method of regulating intestinal microorganisms to affect the function of the nervous system provides a new idea for the treatment of neuralgia. The combination of 16S rRNA gene sequencing and metabolomics can overcome the limitations of single omics to a certain extent and make much progress in the study of the relationship between intestinal microorganisms and health diseases, showing a good application prospect (Goodrich et al., 2016; Wang et al., 2019). In this study, integrated 16S rRNA gene sequencing and metabolomics analysis were used for the first time to study the molecular mechanism of osthole in relieving neuralgia from a new perspective. The purpose of this study is to provide a sufficient theoretical basis and research basis for the application of osthole in the clinical treatment of neuralgia.

The relationship between osthole and gut microbiota and their metabolites is very complicated. On the one hand, osthole could regulate the composition of gut microbiota and catalyze the activity of enzymes produced by metabolites, causing content change, and generation or disappearance of metabolites. On the other hand, gut microbiota participates in the metabolism of osthole, leading osthole to be digested by different gut microbiota. Furthermore, osthole could affect intestinal pH, intestinal transit time, intestinal mucosal function, and so on. The role of gut microbiota in neuralgia has been fully affirmed. The research paper of Myers et al. (2019) indicated that along with astrocytes activation, *Bacteroidetes* and *proteobacteria* increased, while *Firmicutes* decreased in the gastrointestinal of C57BL/6 mice. This result has also been fully affirmed in the research results of Jing et al. (2019), and the characteristics of flora imbalance were that *Bacteroidetes* and *Clostridiales* increased. However, *Firmicutes*, *Lactobacillales*, and *Bifidobacteriales* decreased in the mouse model of spinal cord injury. Zhang et al. (2019) and Lin et al. (2020) found that *Verrucomicrobia*, *Bacteroides*, and *Akkermansia* were increased, whereas *Firmicutes* and *Lachnospira* were decreased in the intestines of patients with neuralgia. The analysis results of 16S rRNA gene sequencing displayed that *Bacteroidetes*, *Firmicutes*, and *Verrucomicrobia* were highly correlated with NP after osthole treatment at the phylum level. *Akkermansia*, *Lachnospiraceae_unclassified*, *Lachnospiraceae_NK4A136_group*, *Bacteroides*, *Lactobacillus*, and *Clostridiales_unclassified* exhibited higher relative abundance and were considered important microbial members at the genus level in neuralgia mice. *Akkermansia* and *Bacteroides* were increased, and *Lachnospiraceae_unclassified*, *Lachnospiraceae_NK4A136_group*, *Lactobacillus*, and *Clostridiales_unclassified* were decreased in Neuralgia mice. The results of this study are consistent with those of previous studies, and there are also some new findings. The relationship between *Clostridiales* and neuralgia has been widely reported in previous studies. Kigerl et al. (2016) reported that *Clostridiales* was increased and *Bacteriodales* was decreased in neuralgia mice, which was different from the viewpoint of Jing et al. (2019). Our results showed that *Clostridiales* did not change significantly in neuralgia mice, whereas *Clostridiales_unclassified* was involved in osthole regulating the neuralgia. Significantly, osthole treatment can change the expression of these gut microbiota in the intestine of CCI mice, indicating a significant role of gut microbiota in the process of osthole relieving neuralgia. The relationship between gut microbiota and NP has attracted more and more clinicians’ attention along with the advancement of medical science. Gut microbiota alleviates the occurrence and development of NP through a variety of pathways, including immune signal pathways (such as chemokines and cytokines, TLRs, and macrophages), neural signal pathway (such as neurotransmitters, TRP channels, microglia, and astrocyte), endocrine and metabolic signaling, etc. The research shows that *Bacteroides* activate TLR5 and promote the release of proinflammatory factors to further enhance the occurrence of neuralgia (Kawai and Akira, 2010). *Lactobacillus* could promote the synthesis of GABA to reverse allodynia in the NP model (Wu and Shah, 2017; Zhao et al., 2017). Also, *Lactobacillus* and *Clostridiales* act on astrocytes through AHR and limit inflammation and neurodegeneration (Zelante et al., 2013; Dodd et al., 2017). How *Akkermansia* and *Lachnospiraceae* alleviate NP is still unclear, but their role in regulating neuralgia is determined. So far, we have formed an overall framework of the relationship between gut microbiota and neuralgia and how osthole alleviates neuralgia through gut microbiota.

Metabolites are inextricably linked with gut microbiota, and intestinal flora metabolites can regulate the brain–gut axis and immune system (Sun and O’Riordan, 2013; Lei et al., 2016; Bridgman et al., 2017). Metabolites will reach the brain through blood circulation as messenger substances and help microglia respond to inflammatory reactions quickly and effectively through the BBB, which indicates that metabolites are likely to be the reason for the continuous flow of information between gut microbiota and microglia in the brain. The lack of some gut microbiota will increase the permeability of the BBB and promote metabolites’ entry into the brain (Al-Asmakh and Hedin, 2015; Kelly et al., 2015). Our results showed that metabolites that belong to sphingolipids, glycerophospholipids, and fatty acyls change in CCI mice. KEGG enrichment analysis of differential metabolites showed that multiple pathways are involved in the process of osthole in treating neuralgia, including the regulation of lipolysis in adipocytes, AGE-RAGE signaling pathway in diabetic complications, and pathogenic *E. coli* infection. Previous investigations indicated that glycerolipids, sphingolipids, glycerophospholipids, and fatty acyls played an essential role in inflammatory diseases (Hecker et al., 2015; Zhao et al., 2018). It is noteworthy that inflammatory factors are mainly released by microglia. There is a bond between metabolites, microglia, and neuralgia, which is also supported by other research. Kiso et al. (2020) reported that a kind of fatty acyls, an exogenous cannabinoid receptor agonist, could exert an analgesic effect in spinal nerve ligation mice. Singh and Spiegel (2020) revealed that the synthesis of sphingolipids is related to bortezomib-induced NP. The dysregulation of Sphingolipids metabolism could increase the expression of S1PR1 in the spinal cord dorsal horn, promoting the occurrence of NP. Hydrolysis of glycerophospholipids could promote the release of polyunsaturated fatty acids and LPC, increasing the temperature activation threshold of TRPM8 and thus an increase in cold sensitivity (Osthues and Sisignano, 2019). It follows that metabolites play an essential role in osthole relieving neuralgia. Gut microbiota affects the occurrence and progression of NP by metabolites. For example, the link between short-chain fatty (SCFA) and neurological diseases, especially chronic pain, has recently been recognized. SCFA acts on its receptor FFAR2/3 and regulates leukocyte functions, such as the production of cytokines (TNF-α, IL-2, IL-6, and IL-10), eicosanoids, and chemokines (such as CCL2) (Vinolo et al., 2011). Therefore, a rough outline of the relationship among gut microbiota, metabolites, and neuralgia was established.

Integrated analysis of metabolomics and gut microbiota could reveal their interaction more deeply. Many gut microbiota and metabolites have a strong positive correlation, such as *Bilophila* and SM 34:1; SM (d14:0/20:1), *Paenochrobactrum* and FA 18:0, PC 36:1; PC (18:0/18:1), FA 22:6, SM 42:3; SM (d14:2/28:1), *Alistipes* and FA 22:6, *Escherichia–Shigella* and FA 18:0, etc. However, *Paenochrobactrum* and LysoPC 18:2 have a strong negative correlation. The correlation of genes, metabolites, and microbial genera displayed that 10 genes were involved in the process of osthole alleviating NP through the metabolic pathway and gut microbiota, including IGF2, GDAP1, MYLK, IL18, CD55, MIR331, FHIT, F3, ERBB4, and ITGB3. The difference in gut microbiota composition between individuals was more than 80%, indicating that genetics play an important role in the formation of gut microbiota (Kurilshikov et al., 2021). Firstly, the difference of specific gene expression determines the composition of gut microbiota and disease state of human bodies, causing metabolites also to be different. Then, gut microbiota metabolites affect the development of disease by regulating different genes. Gut microbiota, metabolites, and genes are inseparable and interact with each other, and play an important role in the development of diseases. The outcome is discussed more deeply from three aspects: gut microbiota, metabolites, and disease genes to reveal the molecular regulation mechanism of metabolites related to gut microbiota. This will help us to understand the complex relationship between gut microbiota, metabolites, and neuralgia at the molecular level.

Although this study showed an overall understanding of which gut microbiota and metabolites changed during NP, there are still some shortcomings. As is known to all, the gut microbiota is a huge population, and they cooperate and influence each other. The effect of single or several bacteria and metabolites on the disease is very limited, and there is still more development space for changing intestinal microorganisms to affect the process of NP. In addition, the molecular mechanism of osthole alleviates NP by gut microbiota has not been reported and needs further research. How gut microbiota affect metabolites also needs further detailed research. Furthermore, we revealed the relationship between gut microbiota, metabolites, and genes in our research. However, the specific molecular mechanism of the interaction between gut microbiota, metabolites, and genes needs to be further verified. If we want to obtain complete and reliable results, we can carry out the following further research. On the one hand, the bioactivity of different gut microbiota and fecal transplantation experiments were carried out. On the other hand, transcriptomics combined with metabolomics studies the specific regulatory mechanism of metabolites. In addition, expand the sample size and conduct more in-depth research combined with clinical data.

## Conclusion

In conclusion, this study preliminarily confirmed that NP is related to gut microbiota disorder. Osthole can affect metabolites by regulating gut microbiota and play a vital role in the treatment of neuralgia. This will lay a foundation for the research and application of osthole in the treatment of neuralgia.

## Data Availability Statement

The original contributions presented in the study are publicly available. This data can be found here: https://www.ncbi.nlm.nih.gov/bioproject/794982 and https://www.ebi.ac.uk/metabolights/MTBLS4098.

## Ethics Statement

The animal study was reviewed and approved by the Animal Experiment Center of Fourth Military Medical University (approval number: XJYYLL-2015612).

## Author Contributions

RL performed the animal experiments. FW and SD analyzed the 16s rRNA data. MY and WZ analyzed the metabolomics’ data. JW wrote the manuscript. All authors read and approved the final manuscript.

## Conflict of Interest

The authors declare that the research was conducted in the absence of any commercial or financial relationships that could be construed as a potential conflict of interest.

## Publisher’s Note

All claims expressed in this article are solely those of the authors and do not necessarily represent those of their affiliated organizations, or those of the publisher, the editors and the reviewers. Any product that may be evaluated in this article, or claim that may be made by its manufacturer, is not guaranteed or endorsed by the publisher.

## Footnotes

1
http://www.omim.org/

2
https://www.drugbank.ca/

3
https://www.disgenet.org/

## Abbreviations

CCI: chronic constriction injury
IL-1β: interleukin-1β
IL-6: interleukin-6
TNF-α: tumor necrosis factor-α
IL-4: interleukin-4
LPS: Lipopolysaccharide
GABA: γ-aminobutyric acid
SCFA: short-chain fatty acids
TLR: Toll like receptors
TRPs: Temperature sensitive transient receptor potential ion channel
JNK: c-JunN-terminal kinase
CXCL1: chemokine (C-X-C motif) ligand 1
CCL2: chemokine (C-C motif) ligand 2
S1PR1: sphingosine-1-phosphate receptor 1
LPC: lysophospholipids like lysophosphatidylcholine
KEGG: Kyoto Encyclopedia of Genes and Genomes
HMDB: The Human Metabolome Database
QC: quantity control
ANOVA: analysis of variance.
