# Supplementary figures and images for: Integrated 16S rRNA Gene Sequencing and Metabolomics Analysis to Investigate the Important Role of Osthole on Gut Microbiota and Serum Metabolites in Neuropathic Pain Mice
Source: Front Physiol. 2022 Feb 7;13:813626. doi: 10.3389/fphys.2022.813626 (PMC8860327; doi:10.3389/fphys.2022.813626)

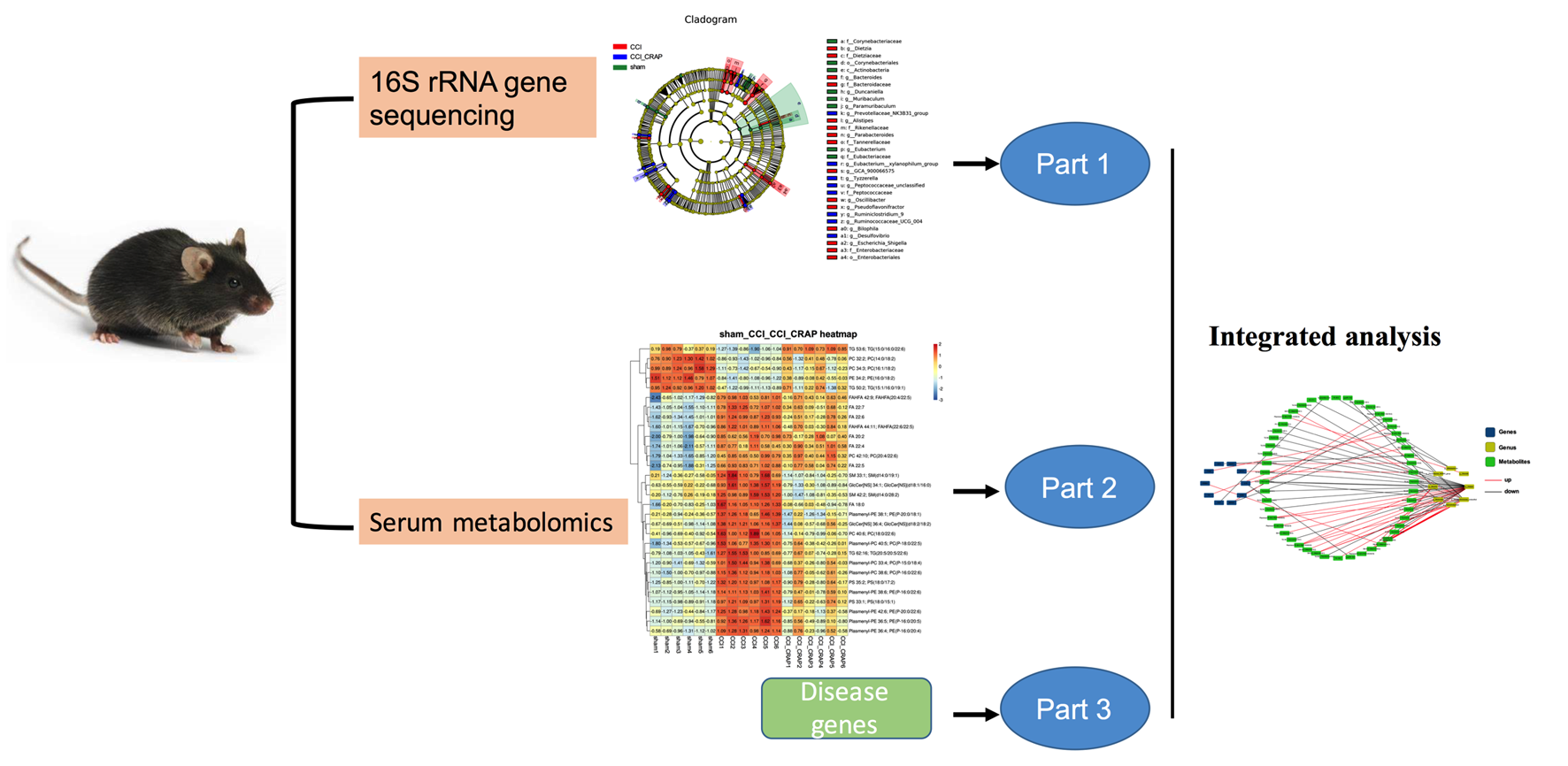

Supplement: Supplementary file 2 [file Image_1.tiff]
